# Nelfinavir: An Old Ally in the COVID-19 Fight?

**DOI:** 10.3390/microorganisms10122471

**Published:** 2022-12-14

**Authors:** Anna Gidari, Samuele Sabbatini, Carlo Pallotto, Sabrina Bastianelli, Sara Pierucci, Chiara Busti, Elisabetta Schiaroli, Daniela Francisci

**Affiliations:** 1 Department of Medicine and Surgery, Clinic of Infectious Diseases, “Santa Maria della Misericordia” Hospital, University of Perugia, 06123 Perugia, Italy; 2 Department of Medicine and Surgery, Medical Microbiology Section, University of Perugia, 06123 Perugia, Italy; 3 Department of Physics and Geology, University of Perugia, 06123 Perugia, Italy

**Keywords:** COVID-19, SARS-CoV-2, molnupiravir, nelfinavir, remdesivir, variant

## Abstract

After almost three years of the pandemic, Severe Acute Respiratory Syndrome Coronavirus 2 (SARS-CoV-2) is still spreading around the world, causing notable sanitary and social issues. New antiviral therapies are constantly under investigation. However, few options have been approved for the treatment of COVID-19. Clinical trials are currently ongoing to evaluate the efficacy of nelfinavir on mild–moderate COVID-19. This study aims to investigate the activity of this compound on SARS-CoV-2 “Variants of Concern” (VOCs), comparing its effectiveness with the approved drugs remdesivir and molnupiravir. The experiments were conducted in a biosafety level 3 facility. In this study, we used a Vero-E6-cell-based infection assay to investigate the in vitro activity of nelfinavir, molnupiravir, and remdesivir. Four strains of SARS-CoV-2 were tested: 20A.EU1, B.1.1.7, P.1, and B.1.617.2. All compounds reached micromolar/submicromolar EC50, EC90, and EC99. Furthermore, the Cmax/EC50 and Cmax/EC90 ratios were >1 for all compounds and all variants tested. Our study demonstrated that nelfinavir, as molnupiravir, and remdesivir are effective in vitro on SARS-CoV-2 variants.

## 1. Introduction

The course of the Coronavirus Disease 2019 (COVID-19) pandemic was improved after the introduction of several efficient vaccines, but the spread of Severe Acute Respiratory Syndrome Coronavirus-2 (SARS-CoV-2) is still occurring due to the emergence of variants of concern (VOCs) [1]. Nowadays, antiviral drugs approved for the treatment of COVID-19 are remdesivir, which is indicated for the treatment of in-hospital patients with pneumonia or as an early treatment of mild–moderate disease (outpatients or inpatients), and molnupiravir and the nirmatrelvir/ritonavir combination, that are indicated for the treatment of patients who are at high risk of progressing to severe COVID-19. The latter are available as oral tablets and have played an important role in the early treatment of the disease, reducing the probability of hospitalization by about 90%. Another orally administrable drug with promising anti-SARS-CoV-2 activity is nelfinavir, a protease inhibitor of human immunodeficiency virus 1 (HIV-1). Clinical trials are now ongoing to demonstrate the efficacy of this drug against COVID-19 [2].

Few studies have demonstrated the potential efficacy of nelfinavir against SARS-CoV-2. Nelfinavir is an antiretroviral drug widely used in the past for the treatment of HIV-1 infection. Its effectiveness is guaranteed by the capacity to selectively inhibit HIV-1 protease preventing cleavage of the gag-pol viral polyprotein that results in the inhibition of virions maturation [3]. Consequently, it has been described how the replication leads to immature and noninfectious virus particles release [4].

In addition, nelfinavir is able to inhibit cell fusion resulting from SARS-CoV-2 Spike (S) glycoprotein. The specific mechanism is not clear, but some studies suggested that its activity is related to the inhibition of the 3CL SARS-CoV-2 main protease. However, Pathak et al. found that nelfinavir seemed to have no activity on this protease while it binds with high-affinity tyrosine-protein-kinase FYN and the neurokinin 2 receptor [5,6]. Furthermore, another study by Narayanan A. et al. showed that nelfinavir does not affect the entry of the virus into the cells [7].

Molnupiravir (EIDD-2801, MK-4482) is a broad-spectrum antiviral drug originally developed for the treatment of influenza and alpha-virus infections. The drug target is the RNA-dependent RNA polymerase (RdRp) that is encoded by all RNA viruses and is the most conserved protein of all RNA viruses [8]. Molnupiravir is administered as a prodrug that is rapidly cleaved in plasma to the active metabolite of the drug [9]. The active metabolite can be used by the RdRp as a substrate to form stable base pairs with a consequent proofreading escape, giving rise to the so-called “viral error catastrophe”. Subsequently, the formation of new complete viral particles is blocked by the mutated products. The broad-spectrum activity of molnupiravir is probably due to this two-step mechanism [10].

Remdesivir is a nucleoside analogue that, as molnupiravir, acts inhibiting the RdRp of coronaviruses. It was originally developed for the treatment of Ebola and, at the beginning of the COVID-19 pandemic, became the first antiviral drug to give successful results on SARS-CoV-2 infection [11]. Remdesivir is a phosphoramidate prodrug that is metabolized to yield an active analogue molecule: remdesivir triphosphate (RTP). The RdRp uses RTP as a substrate, incorporating remdesivir monophosphate (RMP) into the growing RNA product. In coronaviruses, after RMP incorporation, the polymerase can extends the RNA by three more nucleotides before the RNA synthesis stalls [12].

Based on the antiviral mechanism of nelfinavir, molnupiravir, and remdesivir, the emergence of VOCs should not have changed their antiviral activity. To date, the effects of these drugs were mostly evaluated by assessing the cytopathic effect (CPE), cell viability, or by immunofluorescence staining of virions. All these experimental methods generally report SARS-CoV-2 replication or presence without considering the effective infectious ability of viral particles.

Consequently, this study aims to confirm the in vitro efficiency of nelfinavir against the most clinically impacting SARS-CoV-2 VOCs such as B.1.1.7, P.1, and B.1.617.2 in comparison with 20A.EU1 by determining viral infective particles through a plaque assay. Molnupiravir and remdesivir were included in the study in order to compare the effect of nelfinavir with two drugs currently in use in clinical practice.

## 2. Materials and Methods

### 2.1. SARS-CoV-2 Strains Isolation, Vero E6 Cell Cultures, and Antivirals

SARS-CoV-2 strains were manipulated into the Biosafety Level 3 (BSL3) Virology laboratory at “Santa Maria Della Misericordia Hospital”, Perugia, Italy, as previously described [13]. The universal transport medium (UTM) of a nasopharyngeal swab was mixed with a 1:1 nystatin (10,000 U/mL) and a penicillin–streptomycin (10,000 U/mL) solution for 1 h at 4 °C to remove microbial contamination. The mixture was centrifuged at 400× *g* for 10 min and the supernatant was transferred on Vero E6 ATCC CRL-1586 cells monolayer. Cells were cultivated in Eagle’s minimum essential medium (MEM) containing 10% fetal bovine serum (FBS) and 1% (*v*/*v*) penicillin–streptomycin at 37 °C with 5% CO_2_. The supernatant was then titered by a Half-maximal Tissue Culture Infectious Dose (TCID_50_) endpoint dilution assay [14] and stored in small aliquots at −80 °C.

The virus genome sequencing for variant assignation was performed as previously described [15].

Whole-genome sequencing identified a SARS-CoV-2 genome belonging to clade 20A.EU1 (lineage B.1), a B.1.1.7 strain (better known as the Alpha variant), a P.1 strain (also known as the Gamma variant), and a B.1.617.2 strain (also known as the Delta variant) [16]. The SARS-CoV-2 clade 20A.EU1 (lineage B.1) strain was isolated in May 2020 from a symptomatic patient during the first wave of infections. B.1.1.7 and P.1 SARS-CoV-2 clades were isolated in January 2021 during the third wave. Lastly, the B.1.617.2 strain was isolated in July 2021. Virus stock aliquots were thawed immediately before each experiment, diluted to desired concentrations, and discarded after use.

Remdesivir (Veklury^®^, Gilead, Foster City, CA, USA) was suspended in sterile water. Nelfinavir mesylate hydrate (Sigma-Aldrich, St. Louis, MO, USA) and molnupiravir (EID-2801, Sigma-Aldrich, St. Louis, MO, USA) powders were reconstituted in DMSO at a final concentration of 2 mg/mL following the manufacturer’s instructions. Small stock aliquots were prepared and stored at −80 °C. Before each experiment, the compounds were diluted to the desired concentration with complete medium.

### 2.2. Cytotoxicity Assay

Cytotoxic effect of the selected antivirals was obtained by an MTT (3-(4,5-Dimethyl-2-thiazolyl)-2,5-diphenyl-2H-tetrazolium bromide) reduction assay. MTT was purchased from Merck (Merck, Darmstadt, Germany), dissolved in sterile PBS at the concentration of 5 mg/mL, and filter-sterilized immediately before use.

Vero E6 cells (100 µL, 1 × 10^5^/mL) were dispensed into each well of a 96-well plate and incubated at 37 °C with 5% CO_2_ for 24 h. After incubation, the medium was discarded and the monolayers were incubated with 100 µL of nelfinavir (100–0.1 µM), molnupiravir (200–0.1 µM), or remdesivir (200–0.1 µM) for another 24 h in the same conditions. Simple medium and medium with DMSO (0.1–10%) were used as control according to the antiviral solvent used. After treatments, 10 µL of MTT solution were added to each well and the plate was subsequently incubated for 3 h in the same conditions. Finally, 100 µL of DMSO were used to dissolve formazan crystals precipitated on the bottom of the wells after an incubation for 1 h. Absorbance values at 570 nm (reference filter at 630 nm) were obtained using a microplate reader (Tecan Infinite M200, Tecan Trading AG, Mannedorf, Switzerland). The percentages of cytotoxicity of each antiviral were calculated based on cells treated with the respective vehicle (medium with or without DMSO). The latter were used for the determination of the concentrations able to inhibit cells by 50% with respect to the negative control (GI50). The selectivity index (SI), defined as the ratio of GI50 and the 50% effective concentration (EC50), obtained with the following experiments with antivirals, was then calculated. According to the literature, a bioactive drug or biomolecule with an SI ≥ 10 should be further investigated [17].

### 2.3. SARS-CoV-2 Yield Reduction Assay

Vero E6 cells (30,000 cells/well) were cultured in 96-well plates at 37 °C with 5% CO_2_ for 24 h. After incubation, cells were infected with a multiplicity of infection (MOI) of 0.01. Four strains of SARS-CoV-2 were used: B.1 20A.EU1, B.1.1.7, P.1, and B.1.617.2. SARS-CoV-2 strains were allowed to adsorb to the surface of cells for 1 h at 37 °C. Then, the virus inoculum was removed, and cells were treated with 3-fold serial dilutions (0.62–50 µM) of nelfinavir, molnupiravir, and remdesivir. In each plate, negative controls (complete medium and compounds alone) and infected positive controls (SARS-CoV-2 alone) were included. Plates were incubated at 37 °C with 5% CO_2_ for 48 h. After incubation, cell supernatants were stored at −80 °C for subsequent analysis. Furthermore, the plates were stained with 0.5% crystal violet solution for 30 min, then each well was carefully washed to remove dead and nonadherent cells. Crystal violet was solubilized and absorbance at 570 nm was determined by microplate reader.

### 2.4. Plaque-Reduction Assay

The cell supernatants obtained in previous experiments were used for virus titer determination, as previously described [18]. Vero E6 cells (300,000 cells/well) were cultured in 12-well plates for 24 h at 37 °C with 5% CO_2_. The medium of each well was replaced with 250 µL of tenfold serial dilution of supernatants and incubated for 1 h, rocking the plate by hand every 15 min. The supernatants dilutions were removed and 1 mL of overlay medium (complete medium with agar 0.1%) was added to each well for 72 h. After that, the cells were fixed and stained with a solution containing 4% formalin and 0.5% crystal violet. Viral titer was expressed as plaque-forming units (PFU)/mL, considering wells with 2 to 50 plaques. Viral titration was performed in triplicate. The effect of antiviral compounds was expressed as the concentrations able to reduce viral replication by 50, 90, and 99% compared to the control (EC50, EC90, and EC99). Furthermore, the ratio of the maximum achievable plasma concentrations at an approved dose in humans (Cmax) and EC50, EC90, and EC99 values were calculated. Cmax was deduced from literature data. Cmax/(EC50, EC90 or EC99) ratio above 1 was a good indicator of potential human efficacy [19]. Each experiment was performed at least twice. The viral titer reduction was calculated for each compound in comparison to the respective control (sterile water for remdesivir and DMSO for nelfinavir and molnupiravir).

### 2.5. Statistical Analysis and Data Elaboration

Statistical analysis was performed using Prism Graphpad v.8.3 (GraphPad Software, San Diego, CA, USA). Data were tested for normality using the Kolmogorov–Smirnov test and were presented as mean with the respective standard deviation (SD) or median with interquartile range (IQR), as appropriate. EC50, EC90, and EC99 concentrations were calculated using four-parameter variable-slope regression modeling. GI50 values were obtained using nonlinear regression. The Kruskal–Wallis test was used to compare the means of EC50 for each VOC and compound. A *p* value < 0.05 was considered significant.

## 3. Results

Nelfinavir, molnupiravir, and remdesivir were assessed for their cytotoxicity on Vero E6. The cells were treated with different concentrations of each compound and the metabolic activity was evaluated through an MTT assay. The treatment with molnupiravir led to no statistically significant cytotoxic effect at all the tested concentrations, and remdesivir showed a slight reduction of cell viability of about 20% when the concentrations of 100 and 200 µM were used. Conversely, nelfinavir was able to reduce Vero E6 viability by about 70% compared to the control cells only at the highest concentration tested (100 µM). Following these results, the subsequent experiments were performed using the three antivirals at the maximum concentration of 50 µM and with threefold serial dilutions.

**Figure 1:**
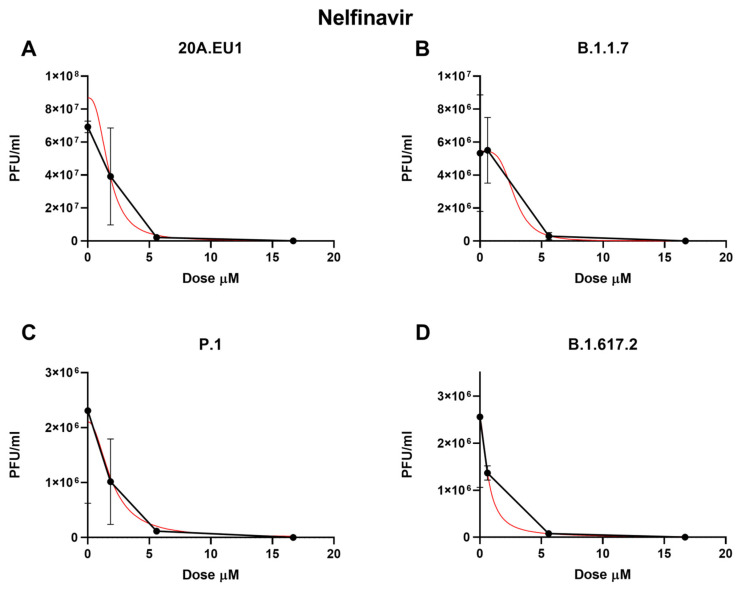
Dose–response inhibition test of nelfinavir (0.62–50 µM) against 20A.EU1 (**A**), B.1.1.7 (**B**), P.1 (**C**), and B.1.617.2 (**D**) strains of SARS-CoV-2 in Vero E6 cells (MOI 0.01). After yield reduction assay on 96-well plates, supernatants were titered with a plaque assay. EC50, EC90, and EC99 were deduced from four-parameter variable-slope regression modeling. The red line represents the nonlinear regression curve. The titers under 10^2^ PFU/mL were excluded from the graphical representation.

The selected compounds were then tested in a yield reduction assay to subsequently determine the number of infectious viral particles on the cell supernatants. Four-parameter variable-slope regression modeling of nelfinavir showed a half-maximal effective concentration (EC50) of 2.00 µM (95% confidence interval, CI 1.18 to 2.83), an EC90 of 3.86 µM, and an EC99 of 7.90 µM (slope of 3.35; 95% CI −5.339 to 12.04) for the 20A.EU1 SARS-CoV-2 strain (Figure 1A and Table 1).

Nelfinavir showed an EC50 of 4.99 µM (the range was too wide for the 95% CI calculation), an EC90 of 5.43 µM, and an EC99 of 5.92 µM (slope of 26.2; the range was too wide for the 95% CI calculation) on the B.1.1.7 strain (Figure 1B). Against P.1, the EC50, EC90, and EC99 were 1.68 µM (95% CI 0.04 to 3.32), 4.17 µM, and 11.25 µM, respectively, with a slope of 2.42 (95% CI −7.87 to 12.7, Figure 1C). Finally, the nelfinavir EC50 for the B.1.617.2 strain was 4.39 µM (the range was too wide for the 95% CI calculation), with an EC90 of 5.12 µM and an EC99 of 6.60 µM (slope of 14.22; the range was too wide for the 95% CI calculation, Figure 1D). The obtained nelfinavir EC50 for the different VOCs were not statistically different (*p* > 0.05). The complete viral inhibition was achieved for all VOCs with a nelfinavir concentration of 50 µM.

**Figure 2:**
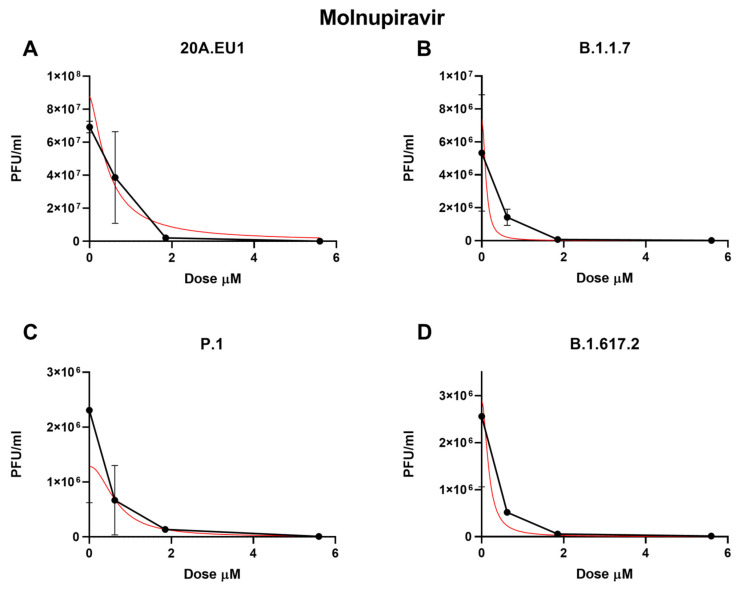
Dose–response inhibition test of molnupiravir (0.62–50 µM) against 20A.EU1 (**A**), B.1.1.7 (**B**), P.1 (**C**), and B.1.617.2 (**D**) strains of SARS-CoV-2 in Vero E6 cells (MOI 0.01). After yield reduction assay on 96-well plates, supernatants were titered with a plaque assay. Effective concentrations (EC50, EC90, and EC99) were deduced from four-parameter variable-slope regression modeling. The red line represents the nonlinear regression curve. The titers under 10^2^ PFU/mL were excluded from the graphical representation.

As shown in Figure 2A and Table 1, the molnupiravir EC50 for the 20A.EU1 SARS-CoV-2 strain was 0.66 µM (95% CI 0.47 to 0.86), and to reach 90% and 99% of viral replication inhibition, the concentrations were increased to 1.27 µM and 2.57 µM, respectively (slope of 3.40; 95% CI 3.25 to 10.04).

Against the B.1.1.7 VOC, molnupiravir showed an EC50 of 0.45 µM (95% CI 0.01 to 1.71), an EC90 of 0.91 µM, and an EC99 of 1.98 µM with a slope of 3.08 (95% CI −23.34 to 29.50, Figure 2B). The P.1 strain was significantly inhibited by the molnupiravir treatment, with an EC50 of 0.37 µM (95% CI 0.01 to 1.25), an EC90 of 1.33 µM, and an EC99 of 5.39 µM (slope of 1.71; 95% CI −5.18 to 8.60, Figure 2C). Finally, 0.33 µM (95% CI 0.01 to 1.59), 0.90 µM, and 2.66 µM of molnupiravir were able to inhibit B.1.617.2 replication by 50%, 90%m and 99%, respectively (slope of 2.21; 95% CI −11.08 to 15.50, Figure 2D). No statistically significant differences between EC50 have been found for the different VOCs (*p* > 0.05). Particularly, the viral replication on the supernatant was not completely inhibited by molnupiravir. Indeed, after the treatment with 50 µM, a residual viral titer was still detected (3.3 to 5 PFU/mL).

**Figure 3:**
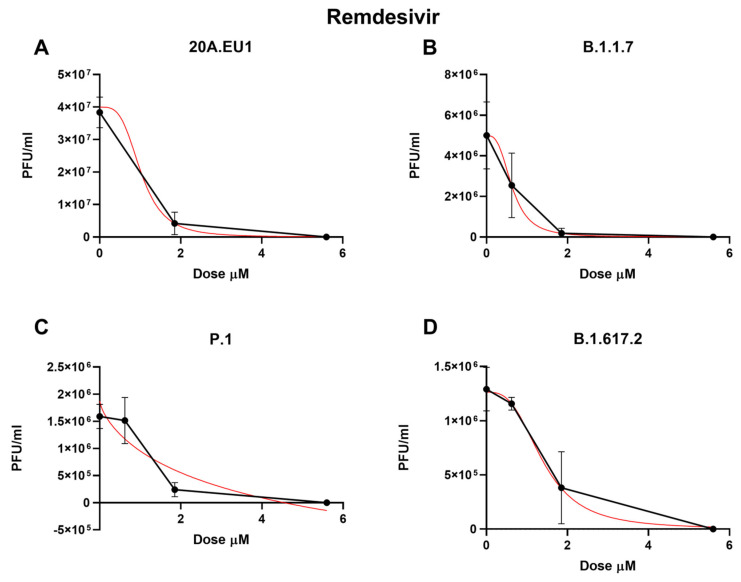
Dose-response inhibition test of remdesivir (0.62–50 µM) against 20A.EU1 (**A**), B.1.1.7 (**B**), P.1 (**C**), and B.1.617.2 (**D**) strains of SARS-CoV-2 in Vero E6 cells (MOI 0.01). After yield reduction assay on 96-well plates, supernatants were titered with a plaque assay. EC50, EC90 and EC99 were deduced from four-parameter variable slope regression modeling. The red line represents the nonlinear regression curve. The titers under 10^2^ PFU/ml were excluded from the graphical representation.

Then, remdesivir was used in our experimental setting to obtain the corresponding effective concentrations. The approximate EC50 for the 20A.EU1 SARS-CoV-2 strain was 1.56 µM (the range was too wide for the 95% CI calculation), for EC90 it was 1.86 µM, and for EC99 it was 2.28 µM, with a slope of 11.91 (the range was too wide for the 95% CI calculation, Figure 3 and Table 1).

Remdesivir activity against VOCs was as follows: on B.1.1.7, it showed an EC50 of 0.63 µM (95% CI 0.42 to 0.83), an EC90 of 1.30 µM, and an EC99 of 2.88 µM (slope of 3.02; 95% CI −3.22 to 9.25); on P.1, the EC50 was 1.24 µM (95% CI 0.70 to 1.79), the EC90 was 2.07 µM, and the EC99 was 3.60 µM (slope of 4.32; 95% CI 0.15 to 8.50); finally, on B.1.617.2, it showed an EC50 of 1.37 µM (95% CI 0.94 to 1.79), an EC90 of 2.97 µM, and an EC99 of 6.92 µM (slope of 2.83; 95% CI 0.88 to 4.78). As for the other antivirals tested, no differences between EC50 have been found for the different VOCs (*p* > 0.05). The complete viral replication inhibition on the supernatant was achieved for all VOCs with a remdesivir concentration of 16.7 µM.

The cytotoxic effects of antiviral on the Vero E6 cells after 48 h of treatment was checked by CV staining. The absorbance values of the treated cells (compounds alone) were not significantly different with respect to the control cells.

The EC50 of each antiviral was used to calculate the SI, as described in the Section 2. The ratios between cytotoxic concentrations and EC50 were >10 for all the antivirals tested (Table 2).

Clearly, it is difficult to translate the in vitro results into clinical practice. There are existing surrogate indexes that could help in this purpose, such as Cmax/EC ratios. To this end, we calculated the Cmax/EC50, Cmax/EC90, and Cmax/EC99 ratios. According to the recent study published by Arshad U. et al., a Cmax/EC50 ratio above 1 is considered a good indicator of potential human efficacy [19].

Based on the literature data, the achievable maximum plasma concentrations are 4 µg/mL (regimen 1250 mg twice daily), 2.97 µg/mL (regimen 800 mg twice daily), and 2.23 µg/mL (regimen 100 mg once daily) for nelfinavir, molnupiravir, and remdesivir, respectively [9,20,21].

As shown in Table 3, the Cmax/EC50 and Cmax/EC90 ratios were above 1 for all compounds against all the VOCs tested. Molnupiravir showed higher values, while remdesivir and nelfinavir demonstrated similar Cmax/EC ratios. In particular, nelfinavir reached Cmax/EC50 ratios between 1.21 and 3.59 for the different variants. The differences found among the variants were not significant for any compound.

Interestingly, the Cmax/EC99 ratio of molnupiravir was above 1 for all VOCs, while for nelfinavir, it was above 1 only for the B.1.1.7 strain, and for remdesivir, the Cmax/EC99 was <1 only for B.1.617.2.

## 4. Discussion

Driven by the results obtained with in vitro experiments and docking-based virtual screening, several molecules have been proposed for COVID-19 treatment. However, few options have been approved by the Food and Drug Administration (FDA) and the European Medicine Agency (EMA) for the treatment of COVID-19. In consideration of the progress of the pandemic and of the importance to treat the SARS-CoV-2 infection early to avoid its evolution into a critical illness, the need for oral antiviral drugs becomes more and more evident. This study aimed to test nelfinavir activity on different VOCs, also comparing their activity with two of the currently available therapies (remdesivir and molnupiravir).

Clinical trials are ongoing to confirm the efficacy of nelfinavir [2]. However, to the best of our knowledge, this drug has not been tested on SARS-CoV-2 variants such as P.1 and B.1.617.2 strains.

Molnupiravir, a new generation broad-spectrum antiviral, has been released with the indication of mild–moderate COVID-19 early treatment [22]. Remdesivir is a nucleoside analogue approved for the treatment of COVID-19 pneumonia and, as a short course of therapy, for mild–moderate SARS-CoV-2 infection [23].

Nelfinavir was found to be the most promising HIV-1 protease inhibitor that could be active on SARS-CoV-2 [24,25]. [In silico studies, such as molecular dynamics simulations, and protease inhibition assays preliminarily suggested an interesting activity of the compound [26,27]. Sixto-Lopez et al. suggested that mutations on protein S may change the viral binding with nelfinavir, but the study could not predict how this finding impacts antiviral activity [28]. In this regard, our study showed how no significant differences in antiviral activity could be found between different strains of SARS-CoV-2.

The effect of nelfinavir in inhibiting viral replication was previously described in our previously published manuscript [25]. In that context, the antiviral was able to prevent cell death to a greater extent than remdesivir treatment. Furthermore, a plaque-reduction assay and real-time PCR analysis showed a significant reduction of viral load after nelfinavir coincubation. It must be considered that the experiments were performed using only the SARS-CoV-2 20A.EU1 strain and with the purpose to analyze the effect of viral infection at the cellular level, but nelfinavir has proven to be a good positive control in limiting the infection and the infection-induced biochemical modifications.

Other in vitro studies on different cell-based experiments confirmed the antiviral potency of this drug, with an EC50 ranging from 0.07 to 3.36 µM [5,6,7,29,30,31].

Of note, the best activity of nelfinavir in reducing the viral titer was obtained by Narayanan et al. by using a plaque assay to determine the EC50. As described above, this result should not be surprising if compared with other methods for the determination of the viral load and considering the mechanism of action of nelfinavir.

These results are not discordant with ours, despite the differences in the experimental protocols. Furthermore, Arshad et al. demonstrated that nelfinavir is active at a low concentration that could be easily reached in vivo. The Cmax/EC90 ratio above 1 should be guaranteed to achieve effective concentrations [19], and our study confirmed these data for all VOCs tested (Cmax/EC90 1.1-1.6).

In addition, the results achieved by cytotoxicity experiments together with data obtained from the antiviral efficacy of nelfinavir showed how effective concentrations of this drug could be easily tolerated by cells. The safety of this molecule may not be new, considering the fact that this drug has been approved and used with the indication for the treatment of another important pandemic such as that of HIV. An added value is certainly given by the selectivity index value obtained by crossing these data with the EC50 of nelfinavir. The latter was found to be much higher than 10 for all the SARS-CoV-2 variants tested. According to the definition, it must be considered that the higher the SI ratio, the theoretically safer and more effective the drug would be during in vivo treatment for a given viral infection.

Other studies also found that nelfinavir could have a synergic activity with other compounds, such as molnupiravir, remdesivir, mefloquine, and others, while alone it is less potent than remdesivir [32]. The efficacy of nelfinavir treatment against SARS-CoV-2 has been also tested in vivo by Foo CS et al. by monitoring hamster infection. The authors found no reduction of viral load in the lung tissue but a substantial improvement in lung pathology [33]. The observed effect could be ascribed to nelfinavir ability to induce the production of noninfectious viral particles, as already described, highlighting the importance of establishing an adequate experimental setting for the assessment of antiviral efficacy.

All these studies agree with the possible role of nelfinavir in the fight against the pandemic and, in this context, the lack of influence of VOCs makes this possibility more concrete. However, the most important study on this topic is the clinical trial ongoing in Japan [2]. It could clarify if nelfinavir deserves a place in COVID-19 therapy. It is important to underline that nelfinavir is an unbranded molecule, so the cost of this therapy would be affordable for every country.

Different in vitro and in vivo studies demonstrated the anti-SARS-CoV-2 activity of molnupiravir. Cox et al., using an in vitro model similar to ours (but with a higher viral dose of infection of 0.1 MOI instead of 0.01), found an EC50 and EC90 of 3.4 μM and 5.4 μM, respectively. Subsequently, they demonstrated that molnupiravir was efficacious to mitigate SARS-CoV-2 infection and block transmission in a ferret model [18].

Molnupiravir was also demonstrated to reduce the viral titer of SARS-CoV-2 in other in vivo models. In particular, in hamsters infected with B.1-G (the Wuhan strain), B.1.1.7, and B.1.351 VOCs, it was able to reduce the titer by 1.8, 2, and 5 logs of TCID_50_/mL [34].

Our study demonstrated that molnupiravir is also active on P.1 and B.1.617.2 in vitro; the latter was one of the most widespread VOCs during the beginning of global vaccine administration. Furthermore, Wahl et al., using a platform based on immunodeficient mice with subcutaneous implantation of human lung tissue, demonstrated the potential activity of molnupiravir as a therapy and as pre-exposure prophylaxis. Furthermore, they found that, probably as expected, the antiviral activity is more powerful the earlier the treatment [35].

Considering these data and the global emergency, there was an unprecedented collaboration between sponsors, contract research organizations, and regulatory authorities that enabled to the acceleration of the passage to in-human studies [36].

Phase 1 studies demonstrated that molnupiravir is safe and well-tolerated and the dose of 800 mg twice daily for 5 days is probably the best choice for posology. For this reason, we considered the Cmax obtained with this dose. According to our data, molnupiravir should reach an effective concentration in vivo [9,37]. In light of the above, molnupiravir received approval for human use and it is now administered as an early therapy for patients at risk of disease progression.

However, it could be metabolized in 2-deoxyribonucleoside that, if embodied in human DNA, could cause mutations as well, as has been shown in mammalian cell cultures (CHO-K1 cells) [38,39]. These data raised concerns about the potential risk of molnupiravir-induced tumorigenesis and teratogenesis that should be further investigated.

According to our data, despite the lower EC50 values obtained, molnupiravir was not able to eradicate SARS-CoV-2 in vitro. This effect could be probably explained by the capacity of the coronaviruses to escape from molnupiravir activity by using a specific exonuclease [40].

Remdesivir has been widely used in COVID-19 treatment and it has been demonstrated in a few studies that its activity is not affected by the emergence of the common variants of concern. However, remdesivir treatment can cause mutations in the RNA-dependent RNA polymerase gene following the treatment, increasing intrahost genomic diversity and resulting in the emergence of novel major variant species [41]. Our study confirmed that remdesivir is active on Alpha, Gamma, and Delta variants and the concentrations reached in vivo are expected to be efficient on these strains.

As shown in Table 3, although the differences identified among variants are not statistically significant, the antiviral effect variability could be explained by the different fitness of each strain. It has been well-described how different variants of SARS-CoV-2 showed lower replication ability and much higher replication times, and these may influence the antiviral response. Indeed, considering that the antiviral acts on a replicant virus, if the replication is slow or ineffective, the antiviral activity could seem lower in vitro.

The limits of this study are the use of in vitro tests, the use of nonhuman cell lines, and the lack of other VOCs, such as Omicron. However, although the Vero E6 cell is not a human cell line, this model is appropriate to test an antiviral compound that does not affect viral entry into the host cells. Indeed, Calu-3 cells, a human cell line obtained from an adenocarcinoma patient, could be more representative because SARS-CoV-2 uses endosomes fusion to enter Vero E6 cells, while lung cells are infected by cell surface fusion. On the Vero E6 model, antivirals against SARS-CoV-2 that target endosomes may not be effective on epithelial cell lines and, consequently, in vivo [42]. Nelfinavir, molnupiravir, and remdesivir, by inhibiting the polymerase activity and the main protease, respectively, could be efficiently tested on Vero E6. Furthermore, we decided to deliberately exclude the most spreading Omicron variants nowadays according to our recently published paper [43]. The less efficient propagation of Omicron VOCs on Vero E6 cells could overestimate/underestimate the antiviral effect obtained in this experimental setting. Above all, the current screening of novel compounds to fight SARS-CoV-2 should also be done with the intent of creating prompt action for future viral pandemics.

## 5. Conclusions

Our study demonstrated that the activities of nelfinavir, molnupiravir, and remdesivir are not affected by the emergence of VOCs. In this context, comparing them with other drugs such as monoclonal antibodies, their activity on VOCs could make the difference. The oral availability of nelfinavir and molnupiravir should permit the early treatment of COVID-19 patients, avoiding disease progression. Furthermore, though the Cmax/EC90 is only an estimate, it indicates a potential achievement of effective concentration in vivo for all the VOCs tested. Following our results, we confirmed that nelfinavir is active against SARS-CoV-2 and its activity results to be very similar to the two drugs used in our work, which, moreover, have been approved and are still in use in the treatment of COVID-19.

The strategy of using old drugs already approved for other pathologies is certainly useful in speeding up the procedures for their approval and use in a short time. In the case of highly prevalent pandemics such as COVID-19, the combined use of in silico approaches and preclinical tests will certainly have their place in future actions against newly emerging pathogens.

## Figures and Tables

**Table 1:** Antiviral effective concentrations on different SARS-CoV-2 strains.

	EC50 (µM)			EC90 (µM)			EC99 (µM)		
	Nel	Mol	Rem	Nel	Mol	Rem	Nel	Mol	Rem
**20A.EU1**	2.00	0.66	1.56	3.86	1.27	1.86	7.90	2.57	2.28
**B.1.1.7**	4.99	0.45	0.63	5.43	0.91	1.30	5.92	1.98	2.88
**P.1**	1.68	0.37	1.24	4.17	1.33	2.07	11.25	5.39	3.60
**B.1.617.2**	4.39	0.33	1.37	5.12	0.90	2.97	6.60	2.66	6.92

Effective concentration (EC), nelfinavir (Nel), molnupiravir (Mol), remdesivir (Rem).

**Table 2:** Selectivity index (SI) of nelfinavir, molnupiravir, and remdesivir.

	SI		
	Nel	Mol	Rem
**20A.EU1**	48.30	ND	946.79
**B.1.1.7**	19.36	ND	2344.44
**P.1**	57.50	ND	1191.13
**B.1.617.2**	22.01	ND	1078.10

Selectivity index (SI); nelfinavir (Nel), molnupiravir (Mol), remdesivir (Rem), Not determined (ND). The SI for molnupiravir has not been determined, since GI50 was higher than the maximum dose tested (200 µM).

**Table 3:** Ratios between the maximum achievable plasma concentrations at an approved dose in humans (Cmax) and EC50, EC90, and EC99 values.

	Cmax/EC50			Cmax/EC90			Cmax/EC99		
	Nel	Mol	Rem	Nel	Mol	Rem	Nel	Mol	Rem
**20A.EU1**	3.01	13.56	2.37	1.56	7.10	1.99	0.76	3.51	1.62
**B.1.1.7**	1.21	20.26	5.90	1.11	9.92	2.85	1.02	4.55	1.29
**P.1**	3.59	24.41	2.97	1.45	6.77	1.79	0.54	1.67	1.03
**B.1.617.2**	1.37	27.16	2.71	1.18	10.04	1.25	0.91	3.39	0.53

Effective concentration (EC), maximum serum concentration (Cmax), nelfinavir (Nel), molnupiravir (Mol), remdesivir (Rem). Cmax and EC are expressed as µg/mL.

## Data Availability

Available on reasonable request.

## References

[1] Gidari A., Schiaroli E., Sabbatini S., Bastianelli S., Pierucci S., Busti C., Francisci D. (2022). Impact of SARS-CoV-2 Omicron variants on serum neutralization in a cohort of healthcare workers vaccinated with BNT162b2. J. Infect..

[2] Hosogaya N., Miyazaki T., Fukushige Y., Takemori S., Morimoto S., Yamamoto H., Hori M., Kurokawa T., Kawasaki Y., Hanawa M. (2021). Efficacy and safety of nelfinavir in asymptomatic and mild COVID-19 patients: A structured summary of a study protocol for a multicenter, randomized controlled trial. Trials.

[3] Yousefi H., Mashouri L., Okpechi S.C., Alahari N., Alahari S.K. (2021). Repurposing existing drugs for the treatment of COVID-19/SARS-CoV-2 infection: A review describing drug mechanisms of action. Biochem. Pharmacol..

[4] Peng C., Ho B.K., Chang T.W., Chang N.T. (1989). Role of human immunodeficiency virus type 1-specific protease in core protein maturation and viral infectivity. J. Virol..

[5] Jan J.T., Cheng T.J.R., Juang Y.P., Ma H.H., Wu Y.T., Yang W.B., Cheng C.W., Chen X., Chou T.H., Shie J.J. (2021). Identification of existing pharmaceuticals and herbal medicines as inhibitors of SARS-CoV-2 infection. Proc. Natl. Acad. Sci. USA.

[6] Pathak N., Chen Y.-T., Hsu Y.-C., Hsu N.-Y., Kuo C.-J., Tsai H.P., Kang J.-J., Huang C.-H., Chang S.-Y., Chang Y.-H. (2021). Uncovering Flexible Active Site Conformations of SARS-CoV-2 3CL Proteases through Protease Pharmacophore Clusters and COVID-19 Drug Repurposing. ACS Nano.

[7] Narayanan A., Narwal M., Majowicz S.A., Varricchio C., Toner S.A., Ballatore C., Brancale A., Murakami K.S., Jose J. (2022). Identification of SARS-CoV-2 inhibitors targeting Mpro and PLpro using in-cell-protease assay. Commun. Biol..

[8] Painter G.R., Natchus M.G., Cohen O., Holman W., Painter W.P. (2021). Developing a direct acting, orally available antiviral agent in a pandemic: The evolution of molnupiravir as a potential treatment for COVID-19. Curr. Opin. Virol..

[9] Painter W.P., Holman W., Bush J.A., Almazedi F., Malik H., Eraut N.C.J.E., Morin M.J., Szewczyk L.J., Painter G.R. (2021). Human safety, tolerability, and pharmacokinetics of molnupiravir, a novel broad-spectrum oral antiviral agent with activity against SARS-CoV-2. Antimicrob. Agents Chemother..

[10] Kabinger F., Stiller C., Schmitzová J., Dienemann C., Kokic G., Hillen H.S., Höbartner C., Cramer P. (2021). Mechanism of molnupiravir-induced SARS-CoV-2 mutagenesis. Nat. Struct. Mol. Biol..

[11] De Clercq E. (2019). New Nucleoside Analogues for the Treatment of Hemorrhagic Fever Virus Infections. Chem. Asian J..

[12] Kokic G., Hillen H.S., Tegunov D., Dienemann C., Seitz F., Schmitzova J., Farnung L., Siewert A., Höbartner C., Cramer P. (2021). Mechanism of SARS-CoV-2 polymerase stalling by remdesivir. Nat. Commun..

[13] Gidari A., Sabbatini S., Bastianelli S., Pierucci S., Busti C., Bartolini D., Stabile A.M., Monari C., Galli F., Rende M. (2021). SARS-CoV-2 Survival on Surfaces and the Effect of UV-C Light. Viruses.

[14] Reed L.J., Muench H. (1938). A simple method of estimating fifty per cent endpoints. Am. J. Epidemiol..

[15] Gidari A., Sabbatini S., Bastianelli S., Pierucci S., Busti C., Monari C., Luciani Pasqua B., Dragoni F., Schiaroli E., Zazzi M. (2021). Cross-neutralization of SARS-CoV-2 B.1.1.7 and P.1 variants in vaccinated, convalescent and P.1 infected. J. Infect..

[16] Lai A., Bergna A., Caucci S., Clementi N., Vicenti I., Dragoni F., Cattelan A.M., Menzo S., Pan A., Callegaro A. (2020). Molecular tracing of SARS-CoV-2 in Italy in the first three months of the epidemic. Viruses.

[17] Indrayanto G., Putra G.S., Suhud F. (2021). Validation of in-vitro bioassay methods: Application in herbal drug research. Profiles of Drug Substances, Excipients and Related Methodology.

[18] Cox R.M., Wolf J.D., Plemper R.K. (2021). Therapeutically administered ribonucleoside analogue MK-4482/EIDD-2801 blocks SARS-CoV-2 transmission in ferrets. Nat. Microbiol..

[19] Arshad U., Pertinez H., Box H., Tatham L., Rajoli R.K.R., Curley P., Neary M., Sharp J., Liptrott N.J., Valentijn A. (2020). Prioritization of Anti-SARS-Cov-2 Drug Repurposing Opportunities Based on Plasma and Target Site Concentrations Derived from their Established Human Pharmacokinetics. Clin. Pharmacol. Ther..

[20] Veklury Assessment Report. European Medicines Agency. https://www.ema.europa.eu/en/documents/assessment-report/veklury-epar-public-assessment-report_en.pdf.

[21] Kattel K., Evande R., Tan C., Mondal G., Grem J.L., Mahato R.I. (2015). Impact of CYP2C19 polymorphism on the pharmacokinetics of nelfinavir in patients with pancreatic cancer. Br. J. Clin. Pharmacol..

[22] Jayk Bernal A., Gomes da Silva M.M., Musungaie D.B., Kovalchuk E., Gonzalez A., Delos Reyes V., Martín-Quirós A., Caraco Y., Williams-Diaz A., Brown M.L. (2021). Molnupiravir for Oral Treatment of COVID-19 in Nonhospitalized Patients. N. Engl. J. Med..

[23] National Institutes of Health COVID-19 Treatment Guidelines Panel. Coronavirus Disease 2019 (COVID-19) Treatment Guidelines. https://www.covid19treatmentguidelines.nih.gov/.

[24] Xie X., Muruato A.E., Zhang X., Lokugamage K.G., Fontes-Garfias C.R., Zou J., Liu J., Ren P., Balakrishnan M., Cihlar T. (2020). A nanoluciferase SARS-CoV-2 for rapid neutralization testing and screening of anti-infective drugs for COVID-19. Nat. Commun..

[25] Bartolini D., Stabile A.M., Bastianelli S., Giustarini D., Pierucci S., Busti C., Vacca C., Gidari A., Francisci D., Castronari R. (2021). SARS-CoV2 infection impairs the metabolism and redox function of cellular glutathione. Redox Biol..

[26] Musarrat F., Chouljenko V., Dahal A., Nabi R., Chouljenko T., Jois S.D., Kousoulas K.G. (2020). The anti-HIV drug nelfinavir mesylate (Viracept) is a potent inhibitor of cell fusion caused by the SARSCoV-2 spike (S) glycoprotein warranting further evaluation as an antiviral against COVID-19 infections. J. Med. Virol..

[27] Avti P., Chauhan A., Shekhar N., Prajapat M., Sarma P., Kaur H., Bhattacharyya A., Kumar S., Prakash A., Sharma S. (2022). Computational basis of SARS-CoV 2 main protease inhibition: An insight from molecular dynamics simulation based findings. J. Biomol. Struct. Dyn..

[28] Sixto-López Y., Correa-Basurto J., Bello M., Landeros-Rivera B., Garzón-Tiznado J.A., Montaño S. (2021). Structural insights into SARS-CoV-2 spike protein and its natural mutants found in Mexican population. Sci. Rep..

[29] Chang C.-W., Parsi K.M., Somasundaran M., Vanderleeden E., Liu P., Cruz J., Cousineau A., Finberg R.W., Kurt-Jones E.A. (2022). A Newly Engineered A549 Cell Line Expressing ACE2 and TMPRSS2 Is Highly Permissive to SARS-CoV-2, Including the Delta and Omicron Variants. Viruses.

[30] Xu Z., Yao H., Shen J., Wu N., Xu Y., Lu X., Zhu W., Li L.-J. (2020). Nelfinavir Is Active Against SARS-CoV-2 in Vero E6 Cells. ChemRxiv.

[31] Ohashi H., Watashi K., Saso W., Shionoya K., Iwanami S., Hirokawa T., Shirai T., Kanaya S., Ito Y., Kim K.S. (2021). Potential anti-COVID-19 agents, cepharanthine and nelfinavir, and their usage for combination treatment. iScience.

[32] Ebisudani T., Sugimoto S., Haga K., Mitsuishi A., Takai-Todaka R., Fujii M., Toshimitsu K., Hamamoto J., Sugihara K., Hishida T. (2021). Direct derivation of human alveolospheres for SARS-CoV-2 infection modeling and drug screening. Cell Rep..

[33] Foo C.S., Abdelnabi R., Kaptein S.J.F., Zhang X., Ter Horst S., Mols R., Delang L., Joana R.-P., Coelmont L., Leyssen P. (2022). HIV protease inhibitors Nelfinavir and Lopinavir/Ritonavir markedly improve lung pathology in SARS-CoV-2-infected Syrian hamsters despite lack of an antiviral effect. Antivir. Res..

[34] Abdelnabi R., Foo C.S., De Jonghe S., Maes P., Weynand B., Neyts J. (2021). Molnupiravir Inhibits Replication of the Emerging SARS-CoV-2 Variants of Concern in a Hamster Infection Model. J. Infect. Dis..

[35] Wahl A., Gralinski L.E., Johnson C.E., Yao W., Kovarova M., Dinnon K.H., Liu H., Madden V.J., Krzystek H.M., De C. (2021). SARS-CoV-2 infection is effectively treated and prevented by EIDD-2801. Nature.

[36] Holman W., Holman W., McIntosh S., Painter W., Painter G., Bush J., Cohen O. (2021). Accelerated first-in-human clinical trial of EIDD-2801/MK-4482 (molnupiravir), a ribonucleoside analog with potent antiviral activity against SARS-CoV-2. Trials.

[37] Khoo S.H., Fitzgerald R., Fletcher T., Ewings S., Jaki T., Lyon R., Downs N., Walker L., Tansley-Hancock O., Greenhalf W. (2021). Optimal dose and safety of molnupiravir in patients with early SARS-CoV-2: A Phase I, open-label, dose-escalating, randomized controlled study. J. Antimicrob. Chemother..

[38] Zhou S., Hill C.S., Sarkar S., Tse L.V., Woodburn B.M.D., Schinazi R.F., Sheahan T.P., Baric R.S., Heise M.T., Swanstrom R. (2021). β-d-N4-hydroxycytidine Inhibits SARS-CoV-2 through lethal mutagenesis but is also mutagenic to mammalian cells. J. Infect. Dis..

[39] Menéndez-Arias L. (2021). Decoding molnupiravir-induced mutagenesis in SARS-CoV-2. J. Biol. Chem..

[40] Wang X., Sacramento C.Q., Jockusch S., Chaves O.A., Tao C., Fintelman-Rodrigues N., Chien M., Temerozo J.R., Li X., Kumar S. (2022). Combination of antiviral drugs inhibits SARS-CoV-2 polymerase and exonuclease and demonstrates COVID-19 therapeutic potential in viral cell culture. Commun. Biol..

[41] Heyer A., Günther T., Robitaille A., Lütgehetmann M., Addo M.M., Jarczak D., Kluge S., Aepfelbacher M., Wiesch J.S.Z., Fischer N. (2022). Remdesivir-induced emergence of SARS-CoV2 variants in patients with prolonged infection. Cell Rep. Med..

[42] White J.M., Schiffer J.T., Bender Ignacio R.A., Xu S., Kainov D., Ianevski A., Aittokallio T., Frieman M., Olinger G.G., Polyak S.J. (2021). Drug Combinations as a First Line of Defense against Coronaviruses and Other Emerging Viruses. mBio.

[43] Gidari A., Sabbatini S., Schiaroli E., Bastianelli S., Pierucci S., Busti C., Comez L., Libera V., Macchiarulo A., Paciaroni A. (2022). The Combination of Molnupiravir with Nirmatrelvir or GC376 Has a Synergic Role in the Inhibition of SARS-CoV-2 Replication In Vitro. Microorganisms.

